# Supplementary material for: RABL6A Promotes Pancreatic Neuroendocrine Tumor Angiogenesis and Progression In Vivo
Source: Biomedicines. 2021 Jun 2;9(6):633. doi: 10.3390/biomedicines9060633 (PMC8228095; doi:10.3390/biomedicines9060633)
Supplement: Supplementary file 1 [file biomedicines-09-00633-s001.zip › biomedicines-1219789 supplement.pdf]

Supplementary Figures

Maharjan et al.

RABL6A promotes pancreatic neuroendocrine tumor angiogenesis and progression *in vivo*

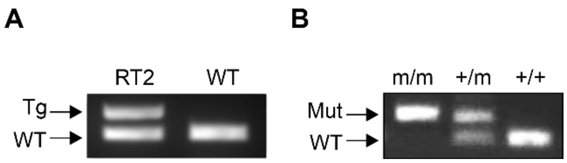

Supplementary Figure 1. Genotyping RIP-Tag2 (RT2) and R6KO mice. **(A)** PCR gel electrophoresis image shows bands representing SV40 large T-antigen (Tg) or a WT DNA segment in RT2 and WT mice. **(B)** Gel images showing bands for WT or mutant *Rabl6* alleles in R6KO (m/m), het (+/m) and WT (+/+) mouse genomic DNA.

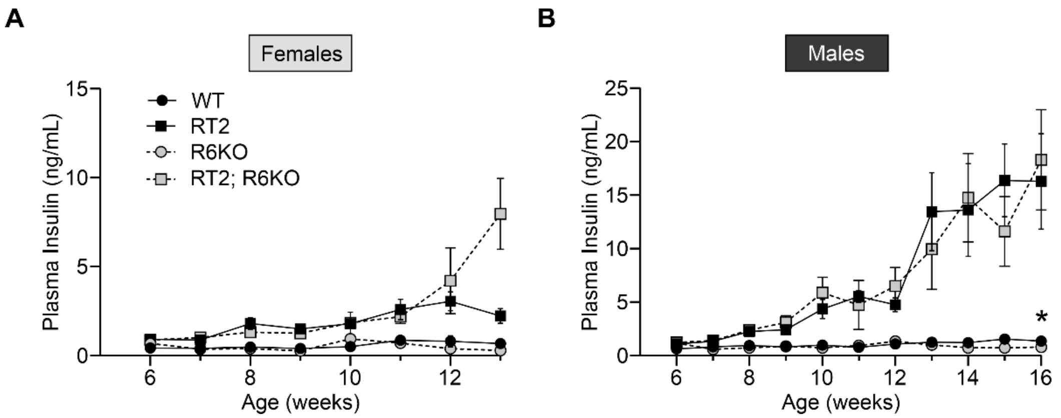

Supplementary Figure 2. Graphs depicting plasma insulin concentration in female (6-13 weeks) and male (6-16 weeks) experimental mice. Plasma insulin was measured by using Mouse Ultrasensitive Insulin ELISA kit from ALPCO®. (A and B) Plasma insulin in WT and R6KO animals is low. WT males have significantly higher plasma insulin concentration than R6KO males (*p<0.05), however, no difference was observed between WT and R6KO females (p = 0.10). Both RT2 and RT2; R6KO females (A) and males (B) exhibit gradual increase in plasma insulin levels as they grow older with no observable difference between the genotypes (p = 0.19 and 0.58 respectively). Data are presented as mean +/- SEM. Linear mixed effects regression models were used for statistical comparison of insulin concentration between groups.

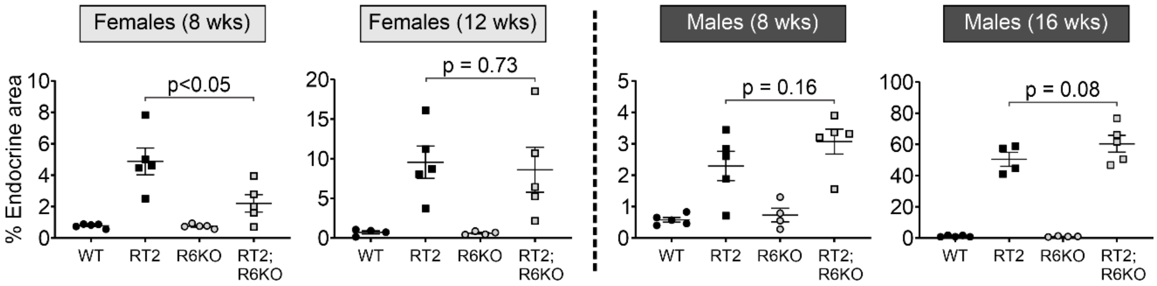

Supplementary Figure 3. Percent endocrine area in the pancreata of female (left) and male (right) experimental mice at the indicated ages. 8-week-old RT2; R6KO females have significantly reduced endocrine area compared to the RT2 controls ($p<0.05$), whereas no difference is seen between the groups at 12 weeks of age ($p = 0.73$). Pancreata of RT2 and RT2; R6KO males at 8 and 16 weeks do not show differences in percent endocrine area ($p = 0.16$ and $p=0.08$, respectively). Data are presented as mean +/- SEM with each dot representing an individual mouse. N = Females: 8 weeks (WT = 5, RT2 = 5, R6KO = 4, RT2; R6KO = 5), 12 weeks (WT = 4, RT2 = 5, R6KO = 4, RT2; R6KO = 5); Males: 8 weeks (WT = 5, RT2 = 5, R6KO = 4, RT2; R6KO = 5), 16 weeks (WT = 5, RT2 = 4, R6KO = 4, RT2; R6KO = 5). Beta regression models were used for statistical analysis.

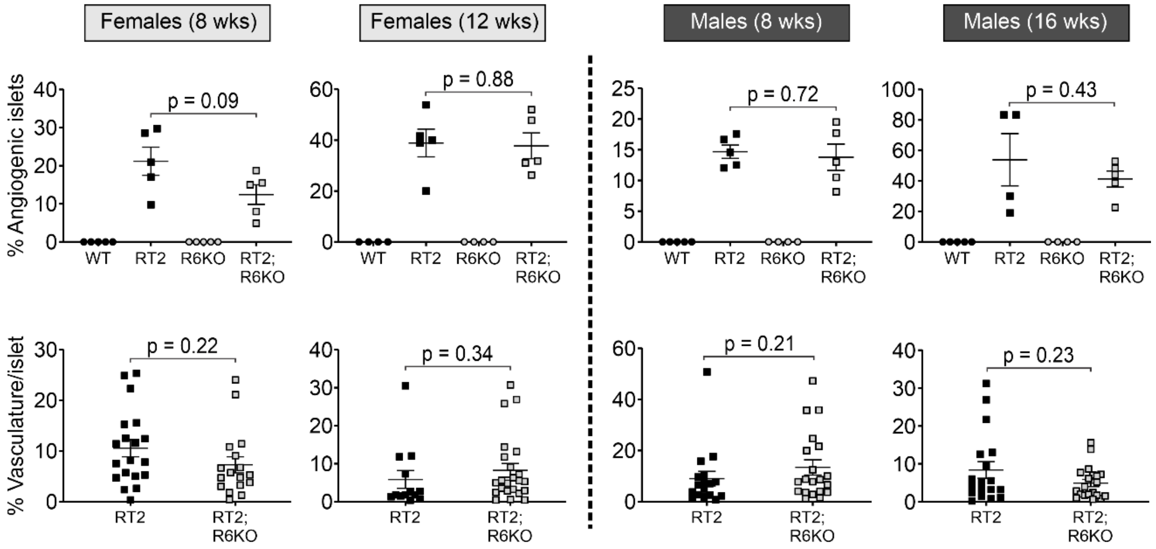

Supplementary Figure 4. Scatterplots showing the percentage of angiogenic islets per pancreas (top row) or percent vascular area per angiogenic islet (bottom row) in female (left) and male (right) mice of the indicated genotypes. Data represent the mean +/- SEM. Each dot represents an individual mouse for endocrine area analyses, whereas an individual islet for vascular area analyses. N = Females: 8 weeks (WT = 5, RT2 = 5, R6KO = 4, RT2; R6KO = 5), 12 weeks (WT = 4, RT2 = 5, R6KO = 4, RT2; R6KO = 5); Males: 8 weeks (WT = 5, RT2 = 5, R6KO = 4, RT2; R6KO = 5), 16 weeks (WT = 5, RT2 = 4, R6KO = 4, RT2; R6KO = 5). Beta regression models were used for statistical analysis.

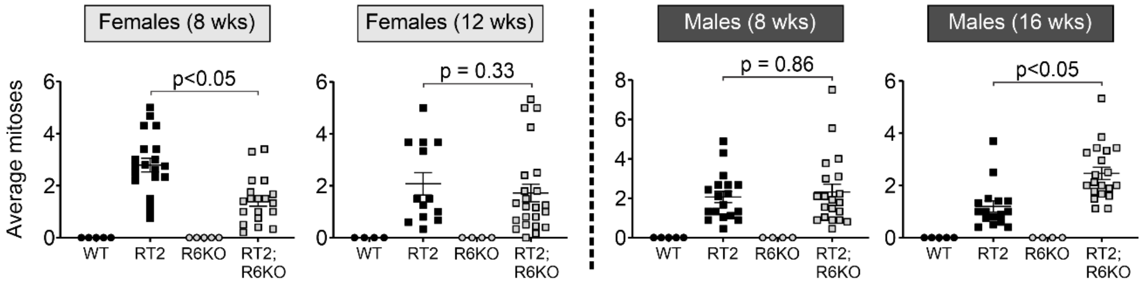

Supplementary Figure 5. Scatterplots depicting the average number of mitoses per field of vision at 600x magnification for individual female (left) and male (right) pancreatic islets. Transformed islets of RT2; R6KO females (8 weeks) have significantly reduced number of mitoses compared to those of RT2 females ($p<0.05$). 16-week-old RT2; R6KO males have higher number of mitoses compared to their RT2 counterparts ($p<0.05$). Data represent the mean +/- SEM. Each dot represents an individual islet: normal islets in WT and R6KO mice versus transformed (hyperplastic, angiogenic, or tumor) islets in RT2 and RT2; R6KO mice. N = Females: 8 weeks (WT = 5, RT2 = 5, R6KO = 4, RT2; R6KO = 5), 12 weeks (WT = 4, RT2 = 5, R6KO = 4, RT2; R6KO = 5); Males: 8 weeks (WT = 5, RT2 = 5, R6KO = 4, RT2; R6KO = 5), 16 weeks (WT = 5, RT2 = 4, R6KO = 4, RT2; R6KO = 5). Linear regression was used to evaluate differences in the average number of mitoses.

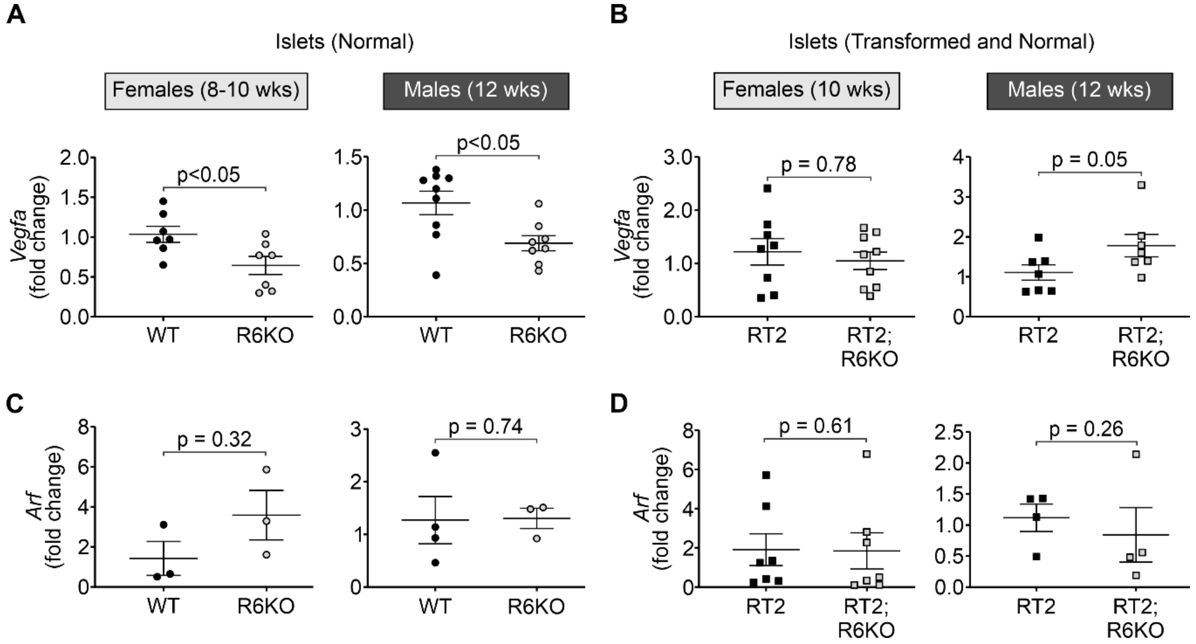

Supplementary Figure 6. Comparison of relative *Vegfa* and *Arf* transcript levels in WT vs R6KO and RT2 vs RT2; R6KO islets isolated from age-matched male and female mice. **(A and B)** Loss of RABL6A reduces *Vegfa* transcript levels in the normal islets of WT males and females, but not in the islets (transformed plus normal mix) of RT2 cohorts. In 12-week-old RT2; R6KO male islets, *Vegfa* mRNA was significantly increased compared to RT2 counterparts. N = Females (WT = 7, RT2 = 8, R6KO = 7, RT2; R6KO = 9); Males (WT = 9, RT2 = 7, R6KO = 8, RT2; R6KO = 7). **(C and D)** No difference in *Arf* mRNA levels were observed for the corresponding age- and sex-matched WT vs R6KO and RT2 vs RT2; R6KO comparisons. N = Females (WT = 3, RT2 = 7, R6KO = 3, RT2; R6KO = 7); Males (WT = 4, RT2 = 4, R6KO = 3, RT2; R6KO = 4). Data are presented as the mean +/- SEM with each dot representing an individual mouse. Linear regression was used to evaluate differences in the transcript levels with p values indicated.
